# Versatile protein tagging in cells with split fluorescent protein

**DOI:** 10.1038/ncomms11046

**Published:** 2016-03-18

**Authors:** Daichi Kamiyama, Sayaka Sekine, Benjamin Barsi-Rhyne, Jeffrey Hu, Baohui Chen, Luke A. Gilbert, Hiroaki Ishikawa, Manuel D. Leonetti, Wallace F. Marshall, Jonathan S. Weissman, Bo Huang

**Affiliations:** 1 Department of Pharmaceutical Chemistry, University of California, San Francisco, California 94143, USA; 2 Tetrad Graduate Program, University of California, San Francisco, California 94143, USA; 3 Department of Cellular and Molecular Pharmacology, University of California, San Francisco, California 94143, USA; 4 Department of Biochemistry and Biophysics, University of California, San Francisco, California 94143, USA; 5 Howard Hughes Medical Institute, San Francisco, California 94143, USA

## Abstract

In addition to the popular method of fluorescent protein fusion, live cell protein imaging has now seen more and more application of epitope tags. The small size of these tags may reduce functional perturbation and enable signal amplification. To address their background issue, we adapt self-complementing split fluorescent proteins as epitope tags for live cell protein labelling. The two tags, GFP11 and sfCherry11 are derived from the eleventh β-strand of super-folder GFP and sfCherry, respectively. The small size of FP11-tags enables a cost-effective and scalable way to insert them into endogenous genomic loci via CRISPR-mediated homology-directed repair. Tandem arrangement FP11-tags allows proportional enhancement of fluorescence signal in tracking intraflagellar transport particles, or reduction of photobleaching for live microtubule imaging. Finally, we show the utility of tandem GFP11-tag in scaffolding protein oligomerization. These experiments illustrate the versatility of FP11-tag as a labelling tool as well as a multimerization-control tool for both imaging and non-imaging applications.

## Abstract (web summary)

Tagging proteins with fluorescent proteins is a powerful method for both imaging and non-imaging applications. Here the authors use the eleventh β-strand of sfGFP and sfCherry as epitope tags for multicolour imaging and amplified signals by tandem arrangement; shortness of the tag enabled introduction into genomic loci using CRISPR/Cas9.

## Graphical abstract

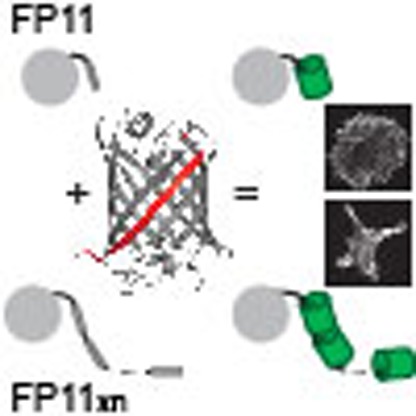

Complementary to direct fluorescent protein fusion, more and more examples of live cell protein labelling and imaging have recently emerged using epitope tags: peptides that do not function by themselves but can be recognized by other intracellularly expressed proteins. An epitope tag fused to the target protein can become an enzyme substrate to be ligated to a small molecule^1^. Alternatively, it can bind an intracellularly expressed antibody/nanobody to bring in functional protein units, such as a fluorescent protein for imaging^2^,^3^ or ubiquitin ligase for protein degradation^4^. This two-component labelling approach has a number of advantages. It is highly modular because the functional units can be easily replaced^2^. A small peptide epitope tag may induce less perturbation than a much larger fluorescent protein. In addition, small epitope tags can be arranged into a multimerization scaffold. For example, the SunTag^2^ allows a dramatic enhancement of fluorescence signal by bringing as many as 24 green fluorescent proteins (GFPs) to a single target protein. This arrangement enables it to successfully overcome an intrinsic problem such as the low affinity between the tagged protein and the binding module. Alternatively, the SunTag could have used a higher affinity and a more precise expression stoichiometry between them, so that there is neither incomplete labelling nor background from excessive binding modules.

One approach to relax this affinity and stoichiometry requirement is to have a binding module that becomes fluorescent only when it is on the epitope. In this case, as long as the binding module is expressed in excess of the target protein, the equilibrium can be shifted towards complete labelling. For this purpose, we adopted a split super-folder GFP (sfGFP) that was previously engineered for efficient self-complementation without the assistance of other protein–protein interactions^5^. This split construct breaks the sequence of sfGFP between the tenth and the eleventh β-strand into two parts: GFP1-10 and GFP11, with GFP11 being a short, 16 amino acid peptide (Fig. 1a). The GFP1-10 fragment, which contains the three residues that constitute the GFP chromophore, is nonfluorescent by itself because chromophore maturation requires the conserved E222 residue located on GFP11 (ref. 6). Upon complementation, the reconstituted GFP becomes fluorescent after the chromophore maturation reaction is completed^5^,^7^. This split GFP system has been previously used for protein quantification^5^, visualization of protein subcellular localization^8^,^9^,^10^, single-molecule imaging^11^, cell-cell contact detection^12^, as well as *in vitro* protein complex assembly^13^. Some of these applications^8^,^9^,^10^ fused the small GFP11 fragment to the target protein as an epitope tag. To expand this approach, here we establish 1-10/11 split fluorescent protein constructs (FP11 tags and the corresponding FP1-10 fragments) as a general method for cellular protein visualization including multicolour imaging, especially for the labelling of endogenous protein via genetic knock-in and signal amplification with tandem tags.

## Results

### Live cell protein labelling using GFP11 and sfCherry11

**Figure 1:**
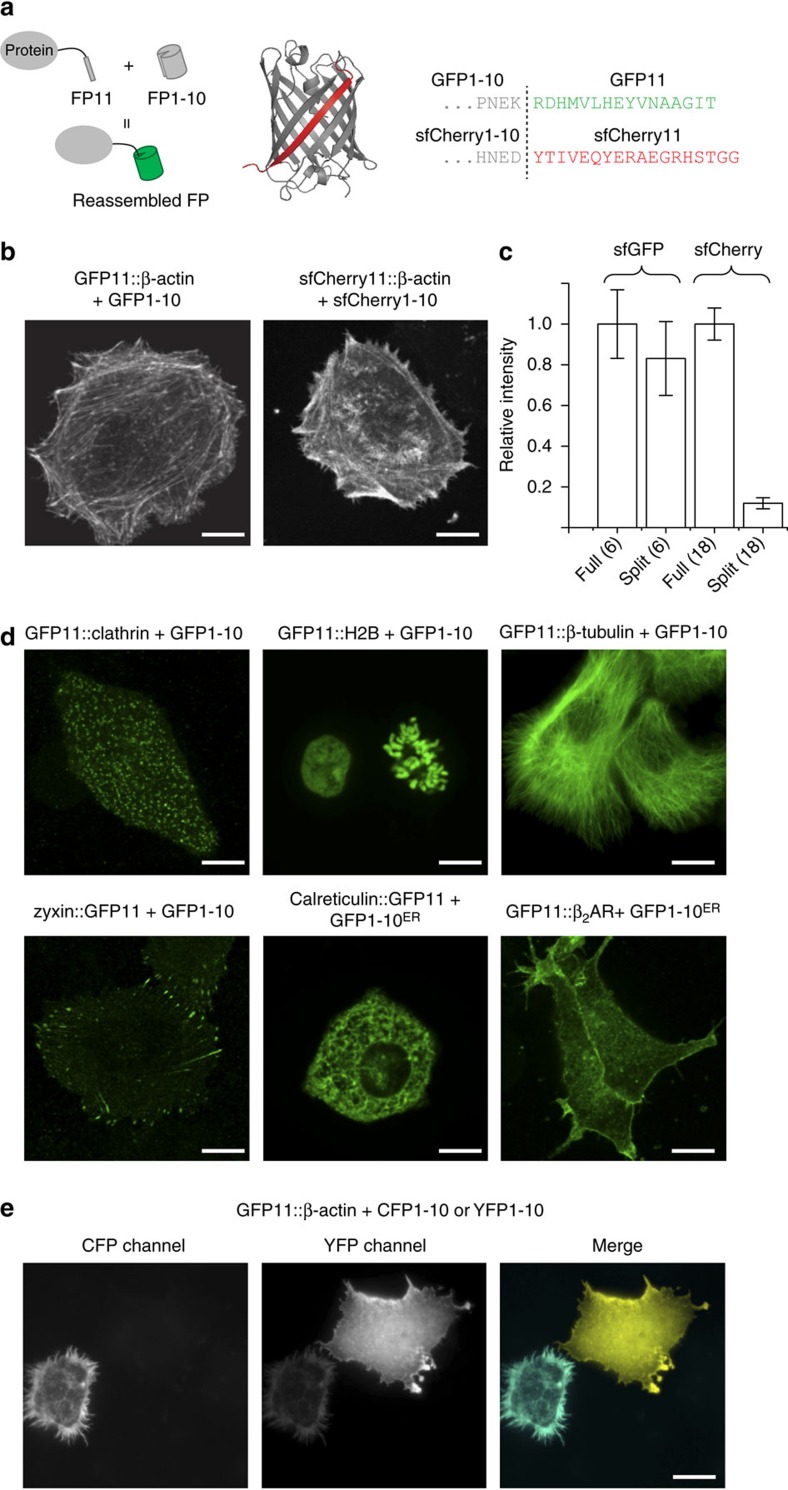
Cellular protein labelling with FP11-tag. (**a**) Schematic diagram for FP11-Tag, illustrated on the crystal structure of sfCherry, and the split schemes. (**b**) Images of HeLa cells co-expressing GFP1-10 and GFP11::β-actin or sfCherry 1-10 and sfCherry 11::β-actin. (**c**) Average fluorescence intensity of whole cells expressing β-actin labelled with full-length sfGFP, sfCherry or the corresponding FP11 tags. *n*=6–18. Error bars are s.e.m. (**d**) Fluorescence images of *Drosophila* S2 cells expressing GFP11-tagged of β-tubulin and calreticulin, and HeLa cells expressing GFP11-tagged clathrin light chain, histone H2B, zyxin and β_2_AR. (**e**) An image of mixed S2 cells expressing either CFP1-10+GFP11::β-actin or YFP1-10+GFP11::β-actin. The weak fluorescence of the CFP1-10-expressing cell in the YFP channel is due to the bleed-through of CFP emission. All the scale bars indicate 5 μm.

To validate protein labelling using FP11-tag in mammalian cells, we fused GFP11 to the (amino) N terminus of human β-actin (with an 18 a.a. GSS linker) and co-expressed it with GFP1-10 in HeLa cells. We observed strong actin-like fluorescence signal, whereas GFP11::β-actin or GFP1-10 alone gave no detectable signal (Fig. 1b and Supplementary Fig. 1). We compared the average fluorescence intensity (representing a combination of expression efficiency, complementation efficiency and fluorophore maturation^14^) of β-actin labelled with either full-length sfGFP or GFP11 and expressed using the same vector (Fig. 1c). The difference between the two labelling approaches was small, indicating that GFP11-tag can label the target protein with a similar efficiency as direct GFP fusion. We then demonstrated that GFP11-tag is compatible with a variety of cellular proteins for live imaging, including cytoskeleton (β-actin and β-tubulin), clathrin-coated pits (clathrin light chain), histone (H2B) and focal adhesion (zyxin) (Fig. 1d). By adding an endoplasmic reticulum (ER) signal peptide to GFP1-10 (GFP1-10^ER^), we were able to specifically label ER luminal protein (calreticulin) or the extracellular domain of plasma membrane protein (β_2_ adrenergic receptor; Fig. 1d). In addition, we showed that the Y66W mutation for cyan fluorescent protein (CFP) and the T203Y mutation for yellow fluorescent protein (YFP)^15^,^16^ can be successfully introduced into GFP1-10 to label proteins with the corresponding colour (Fig. 1e).

To enable multicolour imaging, we tested splitting mCherry at the same site of the GFP1-10/11 system. However, no fluorescent complementation signal was observed when co-expressing mCherry::β-actin and mCherry1-10 (data not shown), which is consistent with previous characterization of split-mCherry constructs^17^. Instead, we were able to obtain a functional split from super-folder Cherry (sfCherry, derived from mCherry for potentially better folding^18^), resulting in an 18 amino-acid peptide for sfCherry11 (Fig. 1a). We verified that sfCherry11::β-actin can be fluorescently imaged when co-expressing sfCherry1-10 (Fig. 1b). Nevertheless, its overall fluorescence signal level is substantially weaker than that of direct sfCherry fusion (Fig. 1c), potentially caused by the less-efficient self-complementation between sfCherry11 and sfCherry1-10. This problem could be solved by further optimization of the split-construct^1^ or by tandem tagging which we will describe later.

### Labelling endogenous proteins with FP11 tag

**Figure 2:**
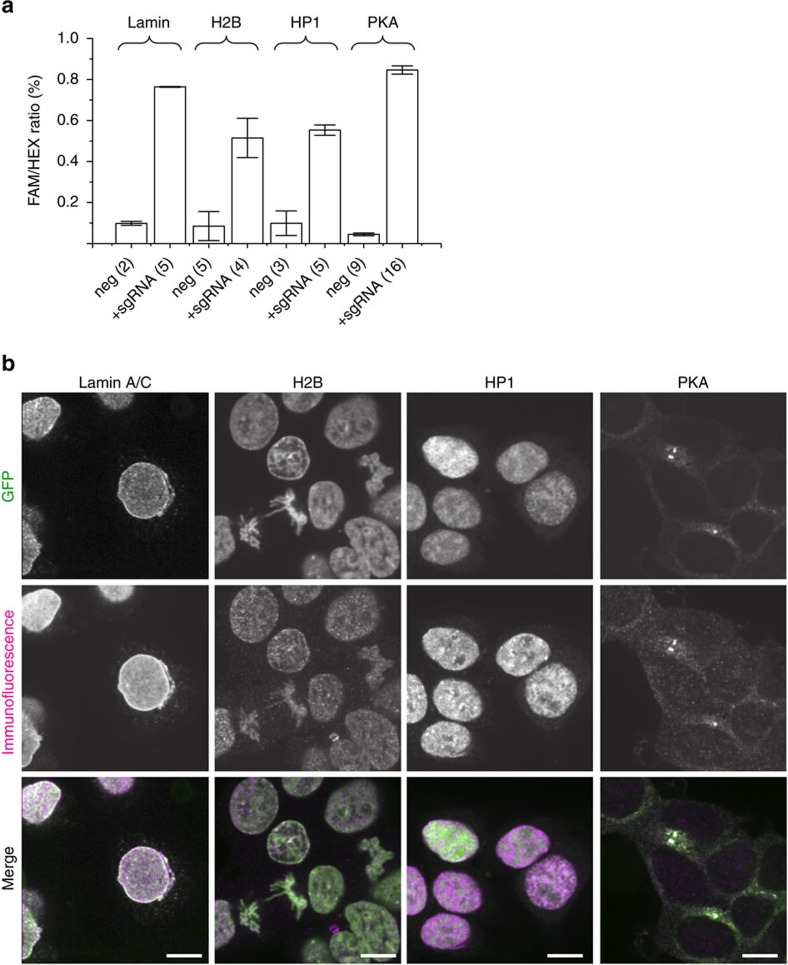
Labelling endogenous proteins using GFP11-tag. (**a**) GFP11 knock-in efficiencies by co-transfection with Cas9/sgRNA expression plasmids and donor templates, quantified by the combined TaqMan PCR/ddPCR assay (see Supplementary Fig. 2). (**b**) GFP fluorescence and immunofluorescence images of knock-in cells. All the error bars are s.e.m. All the scale bars indicate 5 μm. neg, negative.

A first unique application of FP11 epitope tag is to generate libraries of fluorescently labelled endogenous proteins via genetic knock-in by homology-directed DNA repair. Previously, such a library created for *Saccharomyces cerevisiae* has demonstrated its wide application in the systematic investigation of protein localization, gene expression and cellular behaviour^19^,^20^. Because the DNA sequence for GFP11 is only 57 nt (including a 3 a.a. linker), we could use very short homology arms (70 nt on either side, plus the start or stop codon for N- or carboxy (C)-terminal labelling, respectively), so that the entire donor DNA can be a 200 nt single-strand oligo-DNA (ssDNA). Unlike the long donor DNA for full-length GFP, which must be made through multiple cloning steps, GFP11 donor ssDNA can be directly synthesized, thus making it cost-effective for library generation. As a demonstration, we used synthetic ssDNA donors to knock GFP11-tag into four genes, *LMNA*, *HIST2H2BE*, *CBX1* and *PRKACA*, which encode lamin A/C, histone H2B (H2B), heterochromatin protein 1 homologue (HP1) and cAMP-dependent protein kinase catalytic subunit α (PKA), respectively. Using the Cas9/sgRNA plasmid transfection method^21^, we have obtained a knock-in efficiency in the range of 0.5∼0.8% (negative controls: 0.01∼0.09%) (Fig. 2a and Supplementary Fig. 2). Other established knock-in approaches, such as by direct nucleofection of the Cas9/sgRNA complex^22^, may yield higher knock-in efficiencies. We imaged these cells and validated the specificity by immunofluorescence (Fig. 2b). This approach can be readily extended to other mammalian proteins or even animal systems given the recent advancements in CRISPR-mediate genome engineering technologies.

### Signal amplification using tandem FP11 tags

**Figure 3:**
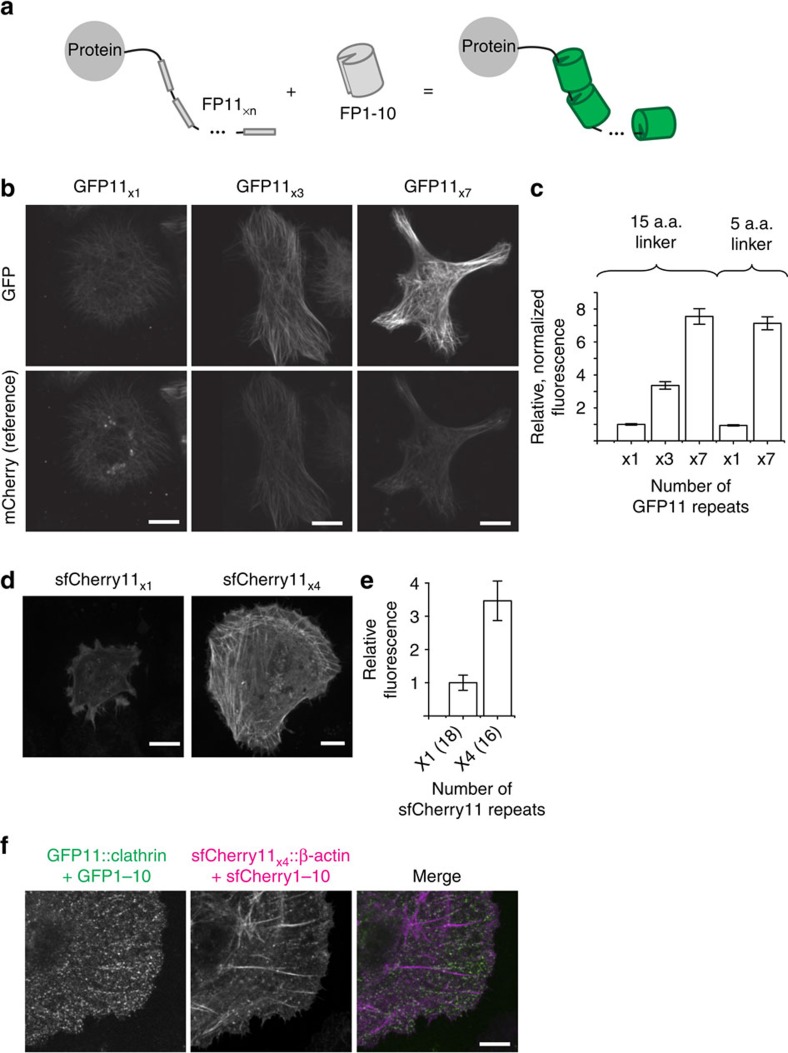
Amplification of fluorescence signal by tandem FP11. (**a**) Schematic of fluorescence signal amplification by tandem FP11 (**b**) Images of *Drosophila* S2 cells expressing GFP11_x1, x3_ or _x7_::mCherry::β-tubulin together with overexpressed GFP1-10. The three panel sets were acquired and displayed with identical settings. (**c**) Quantification of GFP to mCherry fluorescence intensity ratio with different number of GFP11 repeats and different linker lengths between the repeats. Fifty cells were analysed in each case. Error bars are s.e.m. (**d**) Images of HeLa cells expressing sfCherry11_x1_ or _x4_::β-actin together with overexpressed sfCherry1-10. (**e**) Average whole-cell fluorescence intensity with sfCherry11_x1_ or _x4_::β-actin. *n* as indicated in the figure. Error bars are s.e.m. (**f**) Two-colour imaging of GFP11-labelled clathrin light chain and sfCherry11_x4_ labelled β-actin. All the scale bars indicate 5 μm.

With their small sizes, we can arrange FP11-tags into tandem arrays to amplify the fluorescence signal (Fig. 3a), thus addressing the issues of poor signal or photobleaching that live cell imaging often suffers from^23^. To design tandem GFP11_x3_, GFP11_x7_ and sfCherry11_x4_ tags, we purposely used synonymous codons to avoid deleterious recombination during cloning, which can be caused by repetitive nucleic acid sequences. For GFP11_x7_, we tested either 5 a.a. or 15 a.a. linker length between the repeats. Using a fused mCherry as the reference to normalize expression level differences, we observed that the fluorescence signal for GFP11_x3_::mCherry::β-tubulin and GFP11_x7_::mCherry::β-tubulin increased proportionally compared with GFP11::mCherry::β-tubulin without the tandem repeat (Fig. 3b,c). No significant signal difference was recorded for GFP11_x7_ using either the 5 a.a. or 15 a.a. linker (Fig. 3c), indicating a negligible interference between reconstituted GFPs in the tandem tag. We also observed a proportional enhancement of overall cellular fluorescence comparing sfCherry11_x4_::β-actin with sfCherry11::β-actin (Fig. 3d,e). Combining sfCherry11_x4_-tagged β-actin with GFP11-tagged clathrin light chain, we showed two colour imaging using FP11 tags (Fig. 3f). Compared with SunTag^2^, tandem FP11 tags have the advantage that FP1-10 can be overexpressed without causing a fluorescence background, thus allowing a much simpler expression control.

**Figure 4:**
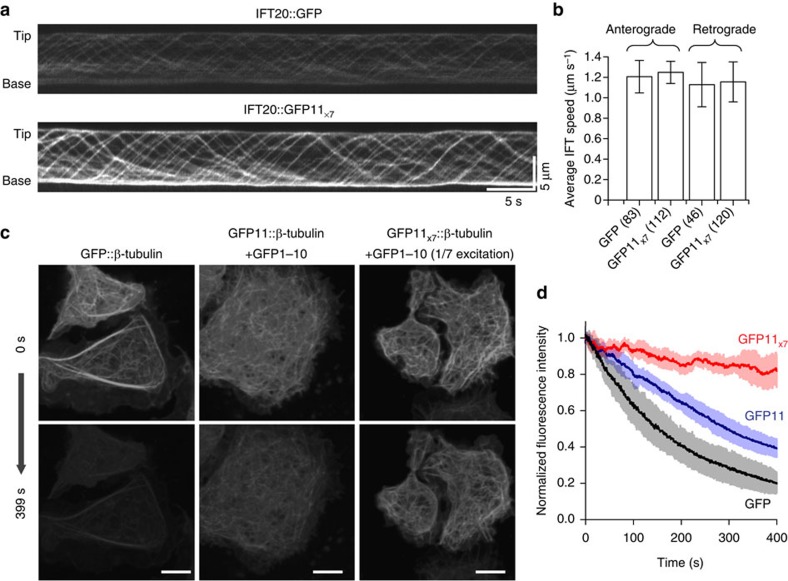
Live cell imaging using tandem FP11 tag. (**a**) Tracking of IFT particles in mouse IMCD3 cells expressing either IFT20::GFP or IFT20::GFP11_x7_+GFP1-10. Kymographs show both retrograde and anterograde transport (see Supplementary Fig. 3 and Supplementary Movie 1). (**b**) Comparison of IFT20 movement speed for IFT20::GFP and IFT20::GFP11_x7_. The number of particles that were analysed for each case is indicated in the figure. Error bars are standard deviations. (**c**) Comparison of the photobleaching rate in imaging β-tubulin labelled with GFP, GFP11 and GFP11_x7_, showing snapshots of S2 cells expressing the three different constructs at the beginning and the end of a 400 s movie. GFP11_x7_ samples were imaged with one-seventh the excitation laser power. Scale bars indicate 5 μm. (**d**) Fluorescence photobleaching time traces. Five cells were averaged in each condition. The error bars are standard deviations.

The fluorescence amplification by tandem FP11-tag can greatly benefit imaging experiments that are difficult because of low signal, such as single particle tracking of intraflagellar transport (IFT) in primary cilia^24^. We fused GFP11_x7_ to the C terminus of IFT20, which moves along ciliary microtubules. Compared with the image of IFT20::GFP, IFT20::GFP11_x7_ generated a much higher signal from IFT particles in the primary cilium of mouse IMCD3 cells (Fig. 4a, Supplementary Fig. 3 and Supplementary Movie 1). Moreover, analysis of the kymograph showed no significant difference between the two labelling methods for either anterograde or retrograde transport speed (Fig. 4a,b), suggesting that IFT20 labelling using tandem GFP11-tag did not perturb its function.

Another advantage of a brighter label is to reduce photobleaching and phototoxicity by lowering excitation intensity while maintaining the same signal level. To demonstrate this application, we imaged *Drosophila* S2 cells expressing GFP::β-tubulin, GFP11::β-tubulin or GFP11_x7_::β-tubulin by spinning disk confocal microscopy (Fig. 4c and Supplementary Movie 2). With one-seventh the excitation laser power, the GFP11_x7_ sample generated a similar level of fluorescence signal compared with either GFP or GFP11, but its photobleaching rate was approximately nine times as slow (Fig. 4d). Surprisingly, even the non-repeating GFP11 displayed approximately three times as slow a photobleaching rate as that of direct GFP fusion. A possible explanation for this effect is that the complementation between GFP11 and GFP1-10 can be reversed, allowing photobleached GFP1-10 to be exchanged for an intact one in the cytoplasm, although such exchange may be limited by the maturation process of the GFP chromophore.

### Tandem FP11 tags as multimerization scaffolds

**Figure 5:**
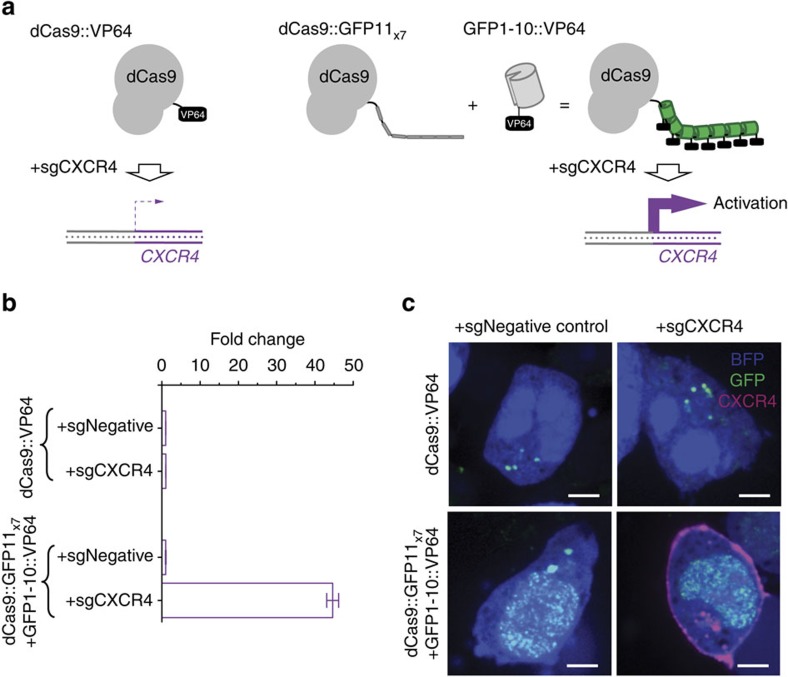
Controlled protein multimerization using GFP11-tag. (**a**) Schematic of *CXCR4* gene activation by either dCas9::VP64 or dCas9::GFP11_x7_+GFP1-10::VP64. (**b**) Plot of CXCR4 fluorescence signals measured by flow cytometry. Only green fluorescence positive cells were analysed, which indicated the expression of both dCas9::GFP11_x7_ and GFP1-10::VP64. Error bars are s.e.m. (**c**) Images of K256 cells infected by lentivirus expressing the two dCas9 constructs with either a targeting or a non-targeting sgRNA. The cells were stained with a fluorescently labelled anti-CXCR4 antibody as an indicator of CXCR4 expression. All the scale bars indicate 5 μm.

Tandem FP11-tag can also be used in non-imaging applications^13^ as a scaffold to assemble multiple copies of proteins for synergistic functioning. Previously, we have developed a CRISPR/Cas9-based transcriptional activation system (CRISPRa)^25^, which activates a specific gene by targeting it with a nuclease-deactivated Cas9 (dCas9) fused to the transcription activator VP64. We have then observed that the activation of natively suppressed gene by CRISPRa often require multiple copies of transcription activation VP64 domains at the target^2^,^26^,^27^,^28^. Here, we showed that tandem GFP11-tag could be used to achieve this goal. We chose to target the C-X-C chemokine receptor type 4 gene *CXCR4*, which is poorly expressed in K265 cells. We fused VP64 to GFP1-10, and then coexpressed it with dCas9::GFP11_x7_ and a *CXCR4* targeting sgRNA^26^ in K265 cells (Fig. 5a). We immunostained these cells for CXCR4 and quantified its expression level by flow cytometry (Fig. 5b). A 45±2-fold signal increase was detected compared with the controls, whereas no significant activation can be observed either with a non-targeting sgRNA or with direct dCas9::VP64 fusion. Immunofluorescence images confirmed CXCR4 expression in these cells (Fig. 5c). In conjugation with SunTag, tandem GFP11 can serve as an orthogonal multimerization tag multiplexed applications. We also observed more robust expression of GFP1-10::VP64, potentially due to its better solubility than scFv::VP64 in SunTag^2^,^26^.

## Discussion

The experiments above showcased the versatility of FP11 as an epitope tag for protein labelling, library generation and signal amplification, in both imaging and non-imaging applications. The functionality of the FP11 tags can be further expanded. For example, we have shown that the point mutations converting GFP to CFP and YFP can be successfully introduced to GFP1-10. Similarly, other functional mutants of GFP, such as blue fluorescent protein (BFP)^15^, photoactivatable GFP (PA-GFP)^29^ and the pH-sensitive pHluorin^30^, may possibly be adaptable for FP11 labelling, too. Of course, as in any protein labelling method, validating the proper functionality of the labelled protein is always essential, especially when tandem GFP11-tag is used. In our case, GFP11_x7_ was well tolerated by multiple proteins including IFT20 and Cas9. Therefore, we expect a broad applicability for tandem GFP11-tag in helping live cell imaging to overcome the issues of poor signal, photobleaching and phototoxicity. We note that when combined with genetic knock-in, the tandem FP11 tag could help the visualization of proteins with low endogenous expression levels. However, knocking in tandem FP11 tags require longer donor ssDNAs that contain the larger insert and potentially longer homology arms. While such long ssDNAs cannot be directly synthesized, they can be produced by reverse transcription methods^31^ to avoid the involvement of cloning.

## Methods

### Molecular cloning

The amino-acid sequence of super-folder mCherry (sfCherry) was obtained from the published literature^18^. sfCherry was split between 208D and 209Y at the middle of the loop between β-strands 10 and 11 (Fig. 1a). For the nucleotide sequence of *sfCherry1-10* and *sfCherry11*, see Supplementary Tables 1 and 2.

The DNAs of *GFP11* (ref. 5) and *sfCherry11* were directly synthesized. The DNAs of H2B, zyxin, clathrin light chain, β-actin were subcloned from the corresponding sfGFP or mEmerald fusion plasmids (cDNA source: the Michael Davidson Fluorescent Protein Collection at the UCSF Nikon Imaging Center). We performed the following restriction enzyme digestion (amino-acid linker length shown in parentheses for each): histone H2B (10 a.a.): *sfGFP* sequence between AgeI and BglII (sfGFP-H2B C-10); zyxin (6 a.a.): *sfGFP* sequence between BamHI and NotI (sfGFP-zyxin-6); clathrin light chain (15 a.a.): *mEmerald* sequence between NheI and BglII (mEmerald-clathrin-15); β-actin (18 a.a.): *mEmerald* sequence between AgeI and BglII (mEmerald-actin-C-18). PCR-amplified *GFP11* as well as *sfCherry11* fragments were then inserted into the digested vectors using In-Fusion assembly (Life Technologies).

For the cloning of GFP11-tagged *Drosophila* calreticulin, *GFP11* was inserted at the nineteenth a.a. position where the signal peptide ends. *GFP11::calreticulin* was inserted into *Drosophila* expression pACUH vectors (source: Yuh-Nung Jan) at the EcoRI/XbaI sites. For the cloning of GFP11-tagged human β_2_AR, the signalling peptide (5HT3R: MRLCIPQVLLALFLSMLTGPGEGS), *β*_*2*_*AR* and *GFP11* were synthesized and cloned into pcDNA3.1 vectors at the BamHI and XhoI sites.

For the expression of GFP1-10, *GFP1-10* was synthesized and cloned into pACUH vectors, pcDNA3.1 vectors as well as modified lentiviral pHR-SFFV vector^32^. For *GFP1-10* sequence information, see Supplementary Table 1. For the expression of *sfCherry1-10*, *sfCherry1-10* was synthesized and cloned into pcDNA3.1 vectors.

To generate an ER-localized GFP1-10 (GFP1-10^ER^), a signal peptide (5′- ATGATGTGGTG CAAAACAGTGATAGTGTTGCTGGCGACAGTCGGCTTTATTAGTGCC -3′) and an ER retention sequence (5′- AGCGAACACGACGAATTG -3′) were fused to the N-terminal and the C-terminal of *GFP1-10*, respectively. The *SP::GFP1-10::SEHDEL* fragment was cloned into pACUH vectors. For mammalian expression, we cloned *SP::GFP1-10* into pcDNA3.1 vectors.

For CFP and YFP imaging, we introduced point mutations in GFP1-10. It has been previously reported that the introduced point mutations Y66W and T203Y in GFP alter the GFP spectral properties to CFP and YFP, respectively. We generated the corresponding mutations in *GFP1-10* with the Q5 site-Directed Mutagenesis Kit (NEB). The primers were designed using the NEB online primer design software (NEBaseChanger). The used primers were as follows: YFP_forward (5′- CTACCTCTCAtatCAAACAGTCCTGAGCAAAGATC -3′), YFP_reverse (5′- TGATTATCAGGAAGAAGTACC -3′), CFP_forward (5′- ACGCTTACGTggGGAGTTCAGTGC -3′), and CFP_reverse (5′- TGTTACGAGAGTCGGCCA -3′). We fused these colour variants to the N terminus of *Drosophila* β-actin and cloned them into pACUH vectors.

To prepare GFP11 repeat arrays constructs, we first synthesized *GFP11*_x1, x3_ or _x7_ with long (15 a.a.) or short (5 a.a.) linkers (see the sequence information in Supplementary Table 2). Then, these GFP11 fragments were fused to β-tubulin, mouse IFT20 and dCas9. Specifically, for the cloning of *GFP11*_x1,x3_ or _x7_::*mCherry*::*β-tubulin*, Drosophila *β-tubulin* (cDNA source: R.Vale) was cloned into pACUH vectors. We then inserted *mCherry*-fused *GFP11*_x1,x3_ or _x7_ at the EcoRI site. For the cloning of *GFP11*_x1_ or _x7_::*Ift20*, we first fused *Ift20* with *GFP11*_x1_ or _x7_, and then we inserted it into the EcoRI/NotI sites of pEGFP-N1 vectors. For the cloning *dCas9*:: *GFP11*_x7_, we modified our SunTag vector (*pHRdSV40-NLS-dCas9-24xGCN4_v4-NLS-P2A-BFP-dWPRE* (addgene #60910)) (ref. 2). *24xGCN4* was cut out by BamHI and NotI, and *GFP11*_x7_ was inserted into these sites.

For the cloning of *sfCherry::β-actin*, we synthesized full-length *sfCherry* and cloned it into the vector containing *β-actin* fragment (see above) at the BglII/NheI site. To construct *sfCherry11*_*x4*_*::β-actin*, we also synthesized *sfCherry11*_*x4*_, and replaced the *sfCherry* fragment to the tandem one at the BglII/NheI site. For the sequence information of *sfCherry11*_*x4*_, see Supplementary Table 2.

For our CRISPRa assay in Fig. 5, we fused an NLS sequence to the C-terminal of *GFP1-10::VP64* and cloned into modified pHR vectors. For the *GFP1-10::VP64::NLS* sequence information, see Supplementary Table 3.

### Cell culture

The cell lines used for imaging were human HeLa (UCSF cell culture facility), HEK293FT (gifted from Bruce Conklin) and K562 cells (UCSF cell culture facility), mouse IMCD 3 cells (UCSF cell culture facility) and *Drosophila* S2 cells (UCSF cell culture facility). HeLa and HEK293FT cells were grown in a Dulbecco's modified Eagle's medium with 10% FCS (UCSF Cell Culture Facility), 100 units ml^−1^ streptomycin, 100 μg ml^−1^ penicillin and 2 mM glutamine. K562 cells were grown in RPMI-1640 with 25 mM HEPES and 2.0 g l^−1^ NaHCo3 in 10% fetal bovine serum (FBS), 100 units ml^−1^ streptomycin, 100 μg ml^−1^ penicillin and 2 mM glutamine. Mouse IMCD3 cells were maintained in a mixture of DMEM and Ham's F12 medium (1:1 vol/vol) with 10% FBS. *Drosophila* S2 cells were grown in Schneider's Drosophila Medium with 10% FBS (Life Technologies).

### Generation of stable cell lines and lentiviral infection

For viral production, HEK293T were plated in T25 flasks. Twenty-four hours later, the 3 μg of lentiviral vector *pHR-SFFV-GFP1-10*, 3 μg of lentiviral packaging plasmid *pCMV-dR8.91* and 0.3 μg of envelop plasmid *pMD2.G* were transfected into the cells using FuGENE HD (Promega) following the manufacturer's protocol. Twenty-four hours after transfection, the culture medium was replaced. Fifty-six hours after transfection, the lentiviral supernatant was harvested and kept at −80 °C for later use. HEK293FT cells stably expressing GFP1-10 were generated by infecting with lentivirus diluted 1:10 in DMEM medium and incubating for 24 h in the medium. We further plated the HEK293FT cells expressing GFP1-10 at one cell per well in a 96-well plate and isolated monoclonal cell lines that showed modest expression.

To construct stable cell lines and measure CRISPRa activity, K562 cells were lentivirally transduced with constructs that express either dCas9::GFP11_x7_ and GFP1-10::VP64 or dCas9::VP64::BFP. The cells were additionally transduced with negative control and CXCR4 targeting sgRNA expression constructs^2^.

For IFT imaging, the plasmids encoding IFT20::GFP (ref. 33) and IFT20::GFP11_x7_ were transfected into IMCD3 cells using Lipofectamine 2000 (Life Technologies) and then selected with Geneticin (400 μg ml^−1^: Life Technologies). After isolating a stable monoclonal cell line, the cells were infected by the GFP1-10 virus-particle-containing medium.

### Transient expression

Mammalian expression plasmids (200 μg of each construct per well) were transfected using Lipofectamine 2000 (Life Technologies) into cultures of HeLa or HEK293FT cells grown on a 48-well plate (Eppendorf). *Drosophila* expression plasmids (100 μg of each construct per well) were transfected using Effectene (QIAGEN) into cultures of S2 cells grown on a 12-well plate (Eppendorf). Note that in Fig. 3 and Supplementary Movie 2, *GFP1-10* and *GFP11*_*x7*_*::mCherry::β-tubulin* plasmids were transfected into S2 cells at a concentration ratio of 7:1. Forty-eight hours after transfection, the cells were live-imaged or fixed with 4% paraformaldehyde for later imaging. To facilitate cilia generation, IMCD3 cells were cultured in serum-free media 1 day before imaging. For two-colour imaging in Fig. 3f, HeLa cells were infected with GFP1-10 lentivirus and, 24 h later, 50 ng of *Clathrin light chain::GFP11*, 100 ng of *sfCherry1-10* and 100 ng of *sfCherry11*_*x4*_*::β-actin* were transfected using Lipofectamine 2000 (Life Technologies).

### Imaging

The cells were grown in 96-well glass bottom plates with #1.5 high-performance cover glass (*In Vitro* Scientific) coated with either Poly-L-Lysine (Sigma-Aldrich) or Fibronectine (Roche) and were imaged on an inverted Nikon Ti-E microscope, Yokogawa CSU-22 confocal scanner unit, × 60/1.4 NA oil objective, an Andor EM-CCD camera (iXon DU897) and Micro-Manager software. Images in Fig. 1e were taken using an inverted Nikon Ti-E microscope with wide-field illumination, × 100/1.4 NA oil objective, and a sCMOS camera (Hamamatsu Flash 4.0). For IFT20 time-lapse imaging in Fig. 4, the cells were grown in DMEM and Ham's F12 (1:1 vol:vol) medium without phenol red, and were imaged in a 37 °C chamber on a Nikon Ti-E microscope with Laser TIRF system, an Andor DU897 EMCCD camera and an Apo TIRF × 100/1.49 NA oil objective. All the imaging experiments were performed at UCSF Nikon Image Center.

### Image analysis

To measure the brightness of full-length FPs and split-FPs in Figs 1c and 3e, we took the images of β-actin-fused full-length FPs or split-FPs. These mCherry, sfCherry or GFP images were taken with × 10 0.3 NA or × 60 1.4 NA objectives, respectively. The acquired images were first background-subtracted; each of an average value of a 10-μm diameter region of interest (ROI) outside a cell was applied for the background subtraction. Then, the mean within a 10-μm diameter ROI inside a cell was calculated for each cell. A total 6∼18 cells were measured for each condition.

To characterize the tandem GFP11-tag, we first set the excitation intensity and exposure time using a GFP11_x1_-linker-mCherry fusion protein (GFP11_x1_::mCherry) so that the camera counts for GFP and mCherry were in the same range. We then imaged the GFP11_x1, x3_ and _x7_ -labelled β-tubulin samples using these parameters. To quantify the ratio of signal enhancement, we acquired images on a wide-field microscope using a × 10/0.3 NA objective to achieve a larger field of view. The acquired images were first background-subtracted and then binarized as ‘0' for outside and ‘1' for inside the cells. The GFP-to-mCherry signal ratio was calculated for each pixel within the mask, and the mean ratio within a 10-μm diameter ROI was computed for each cell. A total 50 cells were measured for each condition.

To trace the fluorescent intensities for live imaging of microtubules in Fig. 4, we used Plot z-axis Profile function in ImageJ. For each photobleaching experiment, we calculated the average fluorescence intensity in a 3 μm × 3 μm square ROI in one cell over the duration of the experiment, subtracted the background measured from a cell-free region in the same field of view, and normalized the trajectory to the average value of the first 10 time points. Figure 4d reported the mean normalized photobleaching trajectories from five experiments for each construct (GFP, GFP11 and GFP11_x7_). For the photobleaching, rates were calculated from the slope of a linear fit to the initial linear segment of the decay curves.

IFT20 moving velocities were measured by kymographs analysis using ImageJ. In brief, we drew a segmented line along a cilium in an image sequence and then created a kymograph using the KymoResliceWide plugin. The velocity of IFP particles were determined by drawing straight lines along IFT trajectories in the kymograph and then measuring the angle of the lines. A detailed particle tracking protocol was described in a previous publication^34^.

### Immunocytochemistry

Mouse monoclonal antibodies used were anti-GFP ([1:100] Sigma-Aldrich, G6539), acetylated tubulin ([1:1,000] Sigma-Aldrich, T7451) and anti-histone H2B ([1:50] Abcam, ab52484) antibodies. Rabbit polyclonal antibodies used were anti-γ-tubulin ([1:1,000] Sigma-Aldrich, T5192), anti-lamin A/C ([1:20] Santa Cruz Biotechnology, H110), anti-cAMP protein kinase catalytic subunit ([1:1,000] Abcam, ab26322) and anti-CBX/HP1 beta antibodies ([1:100] Abcam, ab10478). TRITC or Alexa Fluor 647 conjugated secondary antibodies (Jackson Immuno Research Laboratories, Inc. or Life Technologies, respectively) were used for indirect immunofluorescence detection. BG-conjugated dyes, including SNAP-Surface Alexa Fluor 647 and 546 (NEB), were used for staining SNAP-tag expressed in cells.

The cells were fixed with 4% formaldehyde (Electron Microscopy Sciences) for 15 min, permeabilized with 0.1% Triton X-100 in phosphate-buffered saline (PBS), stained for primary antibodies and Alexa-Fluor-conjugated secondary antibodies, or BG-conjugated fluorophores for SNAP-tag at 4 °C overnight.

### Design of sgRNAs for CRISPR-mediated knock-in

We searched the human genome for CRISPR target sites (GN_20_GG). We used the px330 vector provided through AddGene to generate the sgRNA and Cas9 expression construct. For the individual target sequences, see Supplementary Table 4.

### Design of TaqMan PCR primers and probes

For the design of TaqMan PCR primers and probes, we used the PrimerQuest software (Integrated DNA Technologies) and the Primer-BLAST (NCBI). For the probes, the PrimerQuest default setting gave us optical probe sequences, whereas the primers were identified by the Primer-BLAST. We designed that one of the primers would anneal inside of the donor sequence but not another. The probes and primers were purchased from Integrated DNA Technologies. For sequence of the primers and probes, see Supplementary Table 6.

### Design of homologous recombination templates

To prepare *EGFP-LMNA* homologous recombination templates (70 or 200 bp homology arms), we synthesized two DNA fragments (*LMNA* 5′ regulatory region+one half of a *GFP* coding sequence (1–350 bp), and another half of a *GFP* coding sequence (350–717 bp)+*LMNA* coding region). Subsequently, they were cloned into the pcDNA3.1 vector in which a CMV promoter sequence was removed.

ssDNA donors (200 nt in total length) were manually designed by adding 70 nt homology sequences to both side of the *GFP11* sequence. ssDNA donors were purchased from Integrated DNA Technologies. For the sequence of oligo-nucleotide donors, see Supplementary Table 5.

### CRISPR-mediated knock-in using plasmid transfection

For the knock-in experiments, 200 ng of Cas9+sgRNA vector and 400 ng of an oligonucleotide donor DNA were transfected to HEK293FT cells stably expressing GFP1-10 per 24-well plate (Eppendorf). Genomic DNA was extracted from cells 3 days after transfection with the DNAzol reagent (Molecular Research Center, Inc). We further enriched GFP-positive cells by fluorescence-activated cell sorting.

### Measurement of knock-in efficiency using TaqMan/ddPCR

The premixtures of TaqMan probes and primers consisted of 5 μM of TaqMan probe, 18 μM of a forward primer and 18 μM of a reverse primer. To optimize PCR annealing temperatures and characterize detection efficiencies, we mixed Bio-Rad ddPCR Supermix for Probes (12.5 μl), FAM- and HEX probe and primer premixture (1.25 μl each), and mixture of a genomic DNA (100 ng) and a knock-in allele plasmid (0.5 pg or series of diluted plasmid) in 25 μl total volume. Droplet generation, PCR reaction and droplets read were performed by a QX100 Droplet Generator, a C1000 Thermal Cycler and a QX100 Droplet Reader (Bio-Rad), respectively according to the instructions from the manufacturer. Droplets were analysed by QuantSoft software (Bio-Rad). The optimal annealing temperatures for *LMNA*, *HIST2H2BE*, *CBX1* and *PRKACA* were 62, 60, 60, 59 and 58 °C, respectively.

To measure the knock-in efficiency, we mixed Bio-Rad ddPCR Supermix for Probes (12.5 μl), FAM- and HEX probe and primer premixture (1.25 μl each), and HEK293FT genomic DNA (100 ng) in 25 μl total volume. After droplet generation and PCR reaction, the knock-in frequencies were calculated by taking concentration ratio of a mutant-allele-specific FAM probe and a wild-type-allele-specific HEX probe (*TUBA1A* genomic locus).

### Transcription activation assay

For *CXCR4* gene activation, the cells were dissociated in Gibco Cell Dissociation Buffer and then stained in PBS with 10% FBS for 1 h at room temperature using anti-human CXCR4 antibody (2 μg ml^−1^; Biolegend, 12G5) conjugated to allophycocyanine. *CXCR4* activation was measured using LSR II flow cytometer (BD Biosciences) at 72 or 96 h post infection as written above^25^.

## Additional information

**How to cite this article:** Kamiyama, D. *et al*. Versatile protein tagging in cells with split fluorescent protein. *Nat. Commun.* 7:11046 doi: 10.1038/ncomms11046 (2016).

## Supplementary Material

Supplementary Figures and Supplementary Tables Supplementary Figures 1-3 and Supplementary Tables 1-6

Supplementary Movie 1 Movie of mouse IMCD3 cells expressing either IFT20::GFP (left) or IFT20::GFP11_x7_ + GFP1-10 (right) taken by TIRF microscopy. Images were acquired every 50 ms.

Supplementary Movie 2 Movie of Drosophila S2 cells expressing either GFP::β-tubulin (left), GFP11_x1_::β-tubulin (middle) or GFP11_x7_::β-tubulin (right) taken by confocal microscopy. Images were acquired every 400 ms.
