# Supplementary material for: Versatile protein tagging in cells with split fluorescent protein
Source: Nat Commun. 2016 Mar 18;7:11046. doi: 10.1038/ncomms11046 (PMC4802074; doi:10.1038/ncomms11046)
Supplement: Supplementary Figures and Supplementary Tables — Supplementary Figures 1-3 and Supplementary Tables 1-6 [file ncomms11046-s1.pdf]

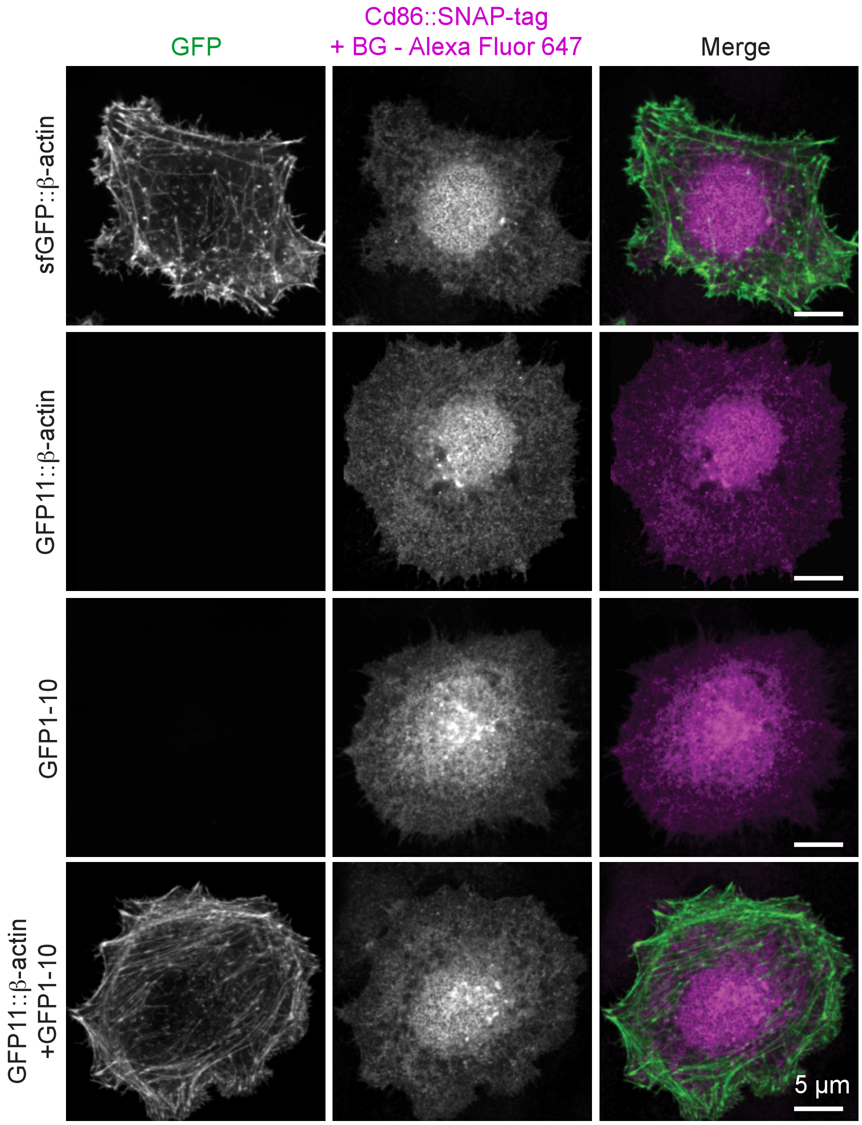

Supplementary Figure 1: Verification of GFP11 labeling, showing images of HeLa cells co-expressing GFP1-10 and GFP11::β-actin, compared to controls with either sfGFP::β-actin, GFP11::β-actin alone or GFP1-10 alone. CD86::SNAP-tag + benzylguanine (BG) - Alexa Fluor 647, which labels the plasma membrane, was used as a transfection marker. Scale bars indicate 5um.

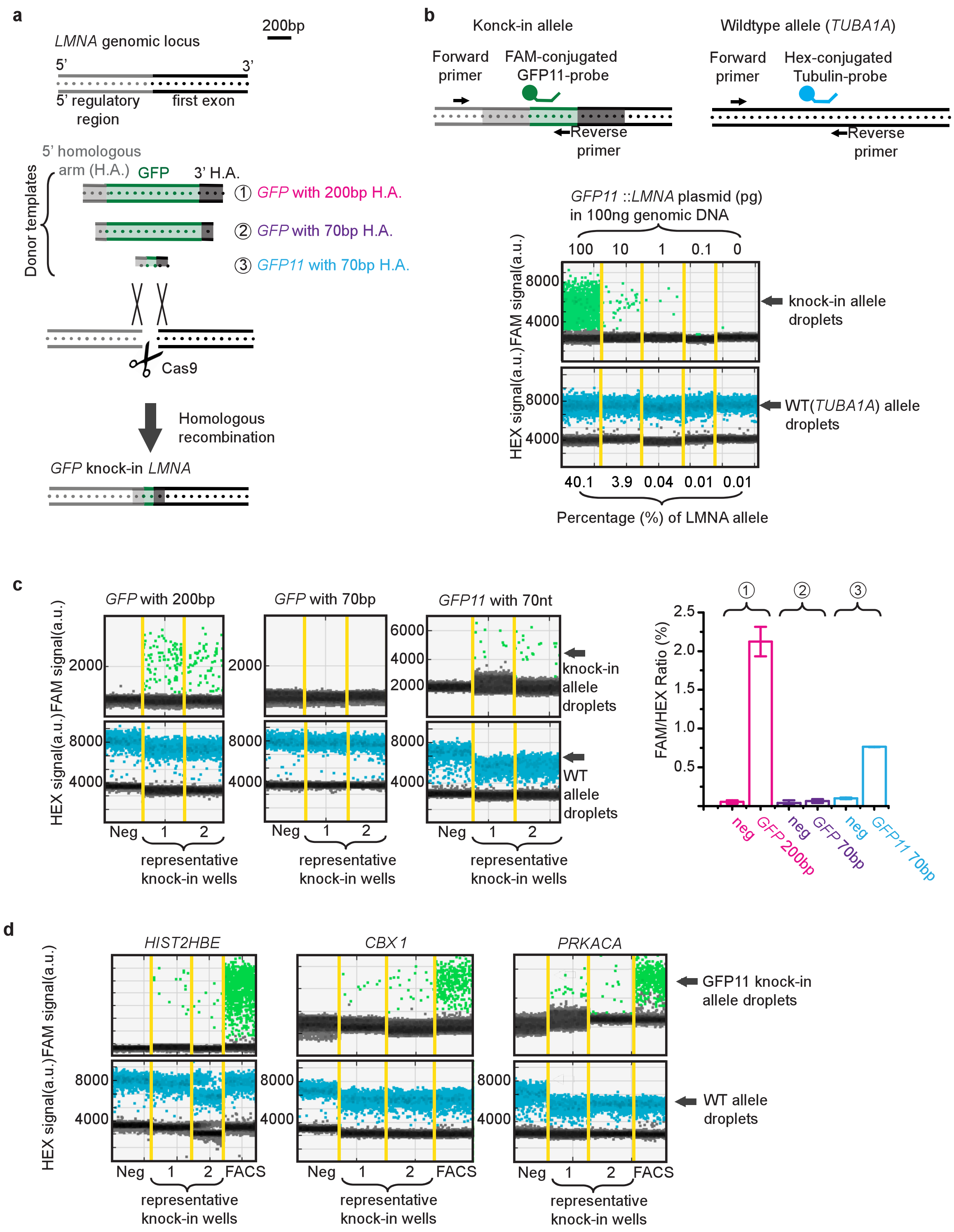

Supplementary Figure 2: TaqManPCR-ddPCR combined assay for knock-in efficiency. (**a**) Schematic for genetic knock-in via CRISPR/Cas9-mediated homologous recombination. Three different donor templates were used to target the same *LMNA*

genomic locus. In addition to GFP11 with 70 nt homology arms (ssDNA), we also tested full length GFP with 200 bp or 70 bp homology arms (double-strand DNA). (**b**) Schematic of TaqManPCR-ddPCR combined assay to quantify the percentage of knock-in alleles. (**c**) ddPCR with indicated amounts of *GFP11::LMNA* plasmid in 100 ng of genomic DNA. Percentages were calculated by taking concentration ratio of a knock-in-allele-specific FAM probe and a wild-type-allele-specific HEX probe. The result indicates that 70 bp homology arm is too short for efficient knock-in of full length GFP using a dsDNA donor. (**c**) ddPCR for *GFP-* or *GFP11*-knock-in alleles at a *LMNA* gene locus. As a negative control, non-targeting sgRNA was used. Quantified data are shown as a bar graph. (**d**) ddPCR for *GFP11*-knock-in alleles at indicated loci. Quantified data are shown in Figure 2a. After enriching GFP$^+$ cells by fluorescence-activated cell sorting (FACS), GFP11-knock-in signal was significantly increased in each case. Dots show droplets containing indicated alleles. Yellow lines indicate the borders between samples.

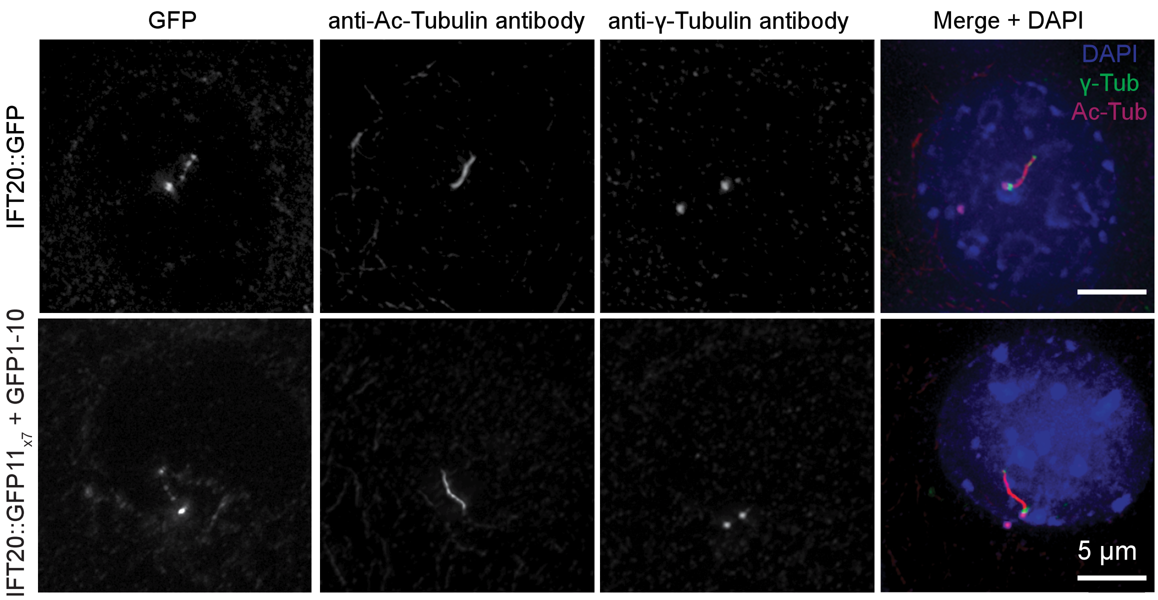

Supplementary Figure 3. Images of mouse IMCD3 cells expressing either IFT20::GFP or IFT20::GFP11$_{x7}$ + GFP1-10 (see Supplementary Movie 1).

Supplementary Table 1: Sequence of *GFP1-10 and sfCherry1-10*

Sequence
GFP1-10<br>ATGTCCAAAGGAGAAGAACTGTTTACCGGTGTTGTGCCAATTTTGGTTGAACTCGATGG<br>TGATGTCAACGGACATAAGTTCTCAGTGAGAGGCGAAGGAGAAGGTGACGCCACCATTG<br>GAAAATTGACTCTTAAATTCATCTGTACTACTGGTAAACTTCCTGTACCATGGCCGACT<br>CTCGTAACAACGCTTACGTACGGAGTTCAGTGCTTTTCGAGATACCCAGACCATATGAA<br>AAGACATGACTTTTTTAAGTCGGCTATGCCTGAAGGTTACGTGCAAGAAAGAACAATTT<br>CGTTCAAAGATGATGGAAAATATAAAACTAGAGCAGTTGTTAAATTTGAAGGAGATACT<br>TTGGTTAACCGCATTGAACTGAAAGGAACAGATTTTAAAGAAGATGGTAATATTCTTGG<br>ACACAAACTCGAATACAATTTTAATAGTCATAACGTATACATCACTGCTGATAAGCAAA<br>AGAACGGAATTAAAGCGAATTTCACAGTACGCCATAATGTAGAAGATGGCAGTGTTCAA<br>CTTGCCGACCATTACCAACAAAACACCCCTATTGGAGACGGTCCGGTACTTCTTCCTGA<br>TAATCACTACCTCTCAACACAAACAGTCCTGAGCAAAGATCCAAATGAAAAA
sfCherry1-10<br>ATGGAGGAGGACAACATGGCCATCATCAAGGAGTTCATGAGATTCAAGGTGCACATGGA<br>GGGCAGCGTGAACGGCCACGAGTTCGAGATCGAGGGCGAGGGCGAGGGCCACCCCTACG<br>AGGGCACCCAGACCGCCAAGCTGAAGGTGACCAAGGGCGGCCCCCTGCCCTTCGCCTGG<br>GACATCCTGAGCCCCCAGTTCATGTACGGCAGCAAGGCCTACGTGAAGCACCCCGCCGA<br>CATCCCCGACTACCTGAAGCTGAGCTTCCCCGAGGGCTTCACCTGGGAGAGAGTGATGA<br>ACTTCGAGGACGGCGGCGTGGTGACCGTGACCCAGGACAGCAGCCTGCAGGACGGCGAG<br>TTCATCTACAAGGTGAAGCTGCTGGGCACCAACTTCCCCAGCGACGGCCCCGTGATGCA<br>GAAGAAGACCATGGGCTGGGAGGCCAGCACCGAGAGAATGTACCCCGAGGACGGCGCCC<br>TGAAGGGCGAGATCAACCAGAGACTGAAGCTGAAGGACGGCGGCCACTACGACGCCGAG<br>GTGAAGACCACCTACAAGGCCAAGAAGCCCGTGCAGCTGCCCGGCGCCTACAACGTGGA<br>CATCAAGCTGGACATCACCAGCCACAACGAGGAC

Supplementary Table 2: Sequences of *GFP11* and *sfCherry11* repeat arrays

Name	Sequence
GFP11 (10aa linker)	ATGCGTGACCACATGGTCCTTCATGAGTATGTAAATGCTGCCGGTATCACCGGGAGTGG<br>TGGGTCCGGCGGGAAATTCATG
GFP11$_{x3}$ (15aa linker)	ATGCGTGACCACATGGTCCTTCATGAGTATGTAAATGCTGCTGGGATTACAGGTGGCGG<br>CGGAAGTGGAGGTGGAGGCTCGGGTGGAGGCAGTTCGCGAGACCATATGGTTCTCCACG<br>AATACGTAAACGCAGCAGGCATCACTGGCGGTGGCGGAAGTGGCGGAGGAGGTTCGGGC<br>GGAGGCAGTTCGCGTGACCATATGGTCCTCCATGAATATGTAAATGCAGCTGGCATTAC<br>TGGAGGTGGCGGTAGTGGCGGAAAATTCATG
GFP11$_{x7}$ (15aa linker)	ATGCGTGACCACATGGTCCTTCATGAGTATGTAAATGCTGCTGGGATTACAGGTGGCTC<br>TGGAAGTTCAGGTGGAGGCTCGGGTGGCGGCAGTTCGAGAGATCATATGGTTCTCCACG<br>AATACGTTAACGCCGCAGGCATCACTGGCAGTGGTGGATCTGGCAGCGGGAGCGGCTCT<br>GGAGGTAGCAGTCGCGACCATATGGTACTACATGAATATGTCAATGCAGCCGGAATAAC<br>CGGATCCGGAAGTGGCTCAAGCGGAGGAGGAAGTAGTGGAAGTTCTCGGGATCACATGG<br>TGCTGCATGAGTATGTGAACGCGGCGGGTATAACTGGTTCGGGAGGCTCAGGTAGCGGC<br>AGTTCAGGAGGAAGCGGGTCCCGAGACCATATGGTGCTTCACGAATACGTAAACGCAGC<br>TGGCATTACTGGGTCAGGAGGTTCAGGAGGGTCTGGTTCTGGATCAGGAGGTAGCAGGG<br>ATCACATGGTACTCCATGAGTACGTGAACGCTGCTGGAATCACAGGCGGTAGCAGTGGT<br>GGAAGTAGCGGCAGCGGCGGCAGTAGCTCACGGGACCATATGGTCCTGCACGAATATGT<br>CAATGCTGCCGGTATCACCGGGAGTGGTGGGTCCGGCGGGAAATTCATG
GFP11 (5aa linker)	ATGCGTGACCACATGGTCCTTCATGAGTATGTAAATGCTGCTGGGATTACAGGTGGCGG<br>CAAATTC
GFP11$_{x7}$ (5aa linker)	ATGCGTGACCACATGGTCCTTCATGAGTATGTAAATGCTGCTGGGATTACAGGTGGCTC<br>TGGAGGTAGAGATCATATGGTTCTCCACGAATACGTTAACGCCGCAGGCATCACTGGCG<br>GTAGTGGAGGACGCGACCATATGGTACTACATGAATATGTCAATGCAGCCGGAATAACC<br>GGAGGGTCCGGAGGCCGGGATCACATGGTGCTGCATGAGTATGTGAACGCGGCGGGTAT<br>AACTGGTGGGTCGGGCGGACGAGACCATATGGTGCTTCACGAATACGTAAACGCAGCTG<br>GCATTACTGGCGGATCAGGTGGCAGGGATCACATGGTACTCCATGAGTACGTGAACGCT<br>GCTGGAATCACAGGCGGTAGCGGCGGTCGGGACCATATGGTCCTGCACGAATATGTCAA<br>TGCTGCCGGTATCACCGGCGGCAAATTCATG
sfCherry11	ATGTACACCATCGTGGAGCAGTACGAGAGAGCCGAGGGCAGACACAGCACCGGCGGC
sfCherry11$_{x4}$ (5aa linker)	ATGTACACAATCGTTGAACAGTACGAGAGGGCAGAGGGTAGGCATAGCACAGGTGGAGG<br>GGGCAGTGGCGGCTACACCATCGTCGAGCAATATGAGAGAGCTGAAGGCCGCCACTCTA<br>CAGGTGGAGGAGGTTCTGGTGGGTATACCATCGTTGAACAGTACGAACGCGCTGAGGGC<br>AGGCACTCCACAGGAGGTGGTGGTAGTGGGGGGTATACCATCGTCGAGCAGTACGAGCG<br>CGCCGAGGGCAGGCACAGTACCGGTGGTGGAGGATCTGGCGGG

GFP11 sequence

sfCherry11 sequence

Linker sequence

Start codon

Supplementary Table 3: Sequence of *GFP1-10::VP64::NLS*

**Sequence**
GFP1-10 ***VP64*** ***NLS***
ATGTCCAAAGGAGAAGAACTGTTTACCGGTGTTGTGCCAATTTTGGTTGAACTCGATGG<br>TGATGTCAACGGACATAAGTTCTCAGTGAGAGGCGAAGGAGAAGGTGACGCCACCATTG<br>GAAAATTGACTCTTAAATTCATCTGTACTACTGGTAAACTTCCTGTACCATGGCCGACT<br>CTCGTAACAACGCTTACGTACGGAGTTCAGTGCTTTTCGAGATACCCAGACCATATGAA<br>AAGACATGACTTTTTTAAGTCGGCTATGCCTGAAGGTTACGTGCAAGAAAGAACAATTT<br>CGTTCAAAGATGATGGAAAATATAAAACTAGAGCAGTTGTTAAATTTGAAGGAGATACT<br>TTGGTTAACCGCATTGAACTGAAAGGAACAGATTTTAAAGAAGATGGTAATATTCTTGG<br>ACACAAACTCGAATACAATTTTAATAGTCATAACGTATACATCACTGCTGATAAGCAAA<br>AGAACGGAATTAAAGCGAATTTCACAGTACGCCATAATGTAGAAGATGGCAGTGTTCAA<br>CTTGCCGACCATTACCAACAAAACACCCCTATTGGAGACGGTCCGGTACTTCTTCCTGA<br>TAATCACTACCTCTCAACACAAACAGTCCTGAGCAAAGATCCAAATGAAAAAgGTTTAA<br>ACGGTAGTGATGCTTTAGACGATTTTGACTTAGATATGCTTGGTTCAGACGCGTTAGAC<br>GACTTCGACCTAGACATGTTAGGCTCAGATGCATTGGACGACTTCGATTTAGATATGTT<br>GGGCTCCGATGCCCTAGATGACTTTGATCTAGATATGCTAGGTAGTactagtGGTGGCG<br>GCGGAAGTCCCAAGAAGAAGCGCAAGGTGTAA

Supplementary Table 4: sgRNA sequences

**sgRNA target**	**DNA sequence**
HIST2H2BE	GTCCCGGCAGGGACTCACT**TGG**
CBX1	GAAAGCTGGCGGGCACTATG**GGG**
LMNA	GCCATGGAGACCCCGTCCCAG**CGG**
PRKACA	GGCCGCCGCCGCCGCGAT**GGG**
ERN1 (non-targeting sgRNA)	GACTCCAGACGCCCTCTGAGCG**AGG**
CxCR4	GCAGACGCGAGGAAGGAGGGCGC
*CxCR4 (*negative control)	GAACGACTAGTTAGGCGTGTA

Guide sequences of 20-25 bases upstream of a PAM sequence (**bold**) were selected.

Supplementary Table 5: Oligo-nucleotide donor DNA sequences

sgRNA target	DNA sequence
HIST2H2BE (200 nt)	GCCCGGCGAGCTGGCCAAGCACGCCGTGTCCGAGGGCACCAAGGCGGTCACCAAGTACA CCAGCTCCAAGGGTGGCGGCCGTGACCACATGGTCCTTCATGAGTATGTAAATGCTGCT GGGATTACATGAGTCCCTGCCGGGACCTGGCGCTCGCTCGCTCGAGTCGCCGGCTGCTT GACTCCAAAGGCTCTTTTCAGAG
CBX1 (200 nt)	CTAATGCCCTTTTTATTTCATTTATCATTTTAGCAGCGTCACCCTTTACACCAGAAAGC TGGCGGGCACTATGCGTGACCACATGGTCCTTCATGAGTATGTAAATGCTGCTGGGATT ACAGGTGGCGGCGGGAAAAAAACAAAACAAGAAGAAAGTGGAGGAGGTGCTAGAAGAGGA GGAAGAGGAATATGTGGTGGAAA
LMNA (200 nt)	TCCTTCGACCCGAGCCCCGCGCCCTTTCCGGGACCCCTGCCCCGCGGGCAGCGCTGCCA ACCTGCCGGCCATGCGTGACCACATGGTCCTTCATGAGTATGTAAATGCTGCTGGGATT ACAGGTGGCGGCGAGACCCCGTCCCAGCGGCGCGCCACCCGCAGCGGGGCGCAGGCCAG CTCCACTCCGCTGTCGCCCACCC
PRKACA (200 nt)	GGGGCCGCCGGCCGCAGCCAGCACCCGCCGCGCCGCAGCTCCGGGACCGGCCCCGGCCG CCGCCGCCGCGATGCGTGACCACATGGTCCTTCATGAGTATGTAAATGCTGCTGGGATT ACAGGTGGCGGCGGCAACGCCGCCGCCGCCAAGAAGGGCAGCGAGCAGGAGAGCGGTGA GTGCCCGGGCTGTGACCCCGATC

GFP11x1 sequence

Linker sequence

Regulatory region sequence

Coding region sequence

Start or stop codon

Supplementary Table 6: TaqMan probes and primers for ddPCR

	Forward primer	GFP11-specific FAM-probe	GFP11-specific Reverse primer
HIST2H2BE ::GFP11	CAAGCGCTCCACCAT CACAT	TGAAGGACCATGTGGTCACG GC	CCCAGCAGCATTTACATA CTCA
GFP11::CBX1	TCCCTGGTTTTCTTA CCATCCA	ATGCGTGACCACATGGTCCT TCAT	CCTGTAATCCCAGCAGCA TTTA
GFP11::LMNA	GGACCTATTAGAGCC TTTGCC	ATGCGTGACCACATGGTCCT TCAT	CCTGTAATCCCAGCAGCA TTTA
GFP11:: PRKACA	CAGCGGCAGAGATCT TGGG	ATGCGTGACCACATGGTCCT TCAT	CCTGTAATCCCAGCAGCA TTTA

	Forward primer	EGFP-specific FAM-probe	EGFP-specific Reverse primer
EGFP::LMNA	GGACCTATTAGAGCC TTTGCC	ATGGTGAGCAAGGGCGAGGA	TCCAGCTCGACCAGGATG

	Forward primer	Wild-type allele specific HEX-probe	Reverse primer
Wild-type allele (*TUBA1A*)	GCGTGAGTGCATCTC CAT	CTCTACTGCCTGGAACACGG CATC	ACTTGGCATCTGGCCATC
